# The Epilepsy‐Desirability of Outcome Ranking (DOOR) as a Multi‐Faceted Consumer‐Informed Outcome Measure for Epilepsy Clinical Trials

**DOI:** 10.1111/ene.70531

**Published:** 2026-02-10

**Authors:** Lucy Vivash, Hannah Johns, Terence J. O′Brien, Leonid Churilov

**Affiliations:** ^1^ Department of Neuroscience, School of Translational Medicine Monash University Melbourne Australia; ^2^ Department of Neurology Alfred Health Melbourne Australia; ^3^ Department of Medicine (Royal Melbourne Hospital) University of Melbourne Melbourne Australia; ^4^ Department of Neurology Royal Melbourne Hospital Parkville Australia

**Keywords:** consumer design, epilepsy, interventional clinical trial, trial outcomes

## Abstract

**Background:**

New drug trials in drug resistant epilepsy are typically powered to detect changes in seizure frequency as the primary endpoint, without integrating other treatment‐associated benefits and harms. We developed Epilepsy‐DOOR, a consumer‐codesigned outcome measure, which combines seizure frequency with quality of life and adverse event measures. This study evaluated Epilepsy‐DOOR in previously completed phase 3 clinical trials of adjunctive brivaracetam in patients with drug resistant epilepsy.

**Methods:**

Epilepsy‐DOOR was derived for each participant who completed the randomised controlled trial of three Phase 3 trials of brivaracetam (N01252, N01253, N01254). Win odds were estimated for Epilepsy‐DOOR and its individual components: change in seizure frequency, quality of life, and adverse event severity for treatment with brivaracetam over placebo for each dose in each study. Odds ratio was estimated for responder rate.

**Results:**

In N01252, Epilepsy‐DOOR demonstrated benefit of 100 mg brivaracetam (Win odds 1.42, 95% CI 1.03, 1.97) but not smaller doses over placebo, in line with the results when using responder rate (odds ratio 1.14, 95% CI 1.02, 1.29). In studies N01253 and N01254, benefit as assessed by Epilepsy‐DOOR did not attain statistical significance, despite benefits at some doses when measuring seizure responder rate.

**Conclusion:**

Epilepsy‐DOOR showed similar effect sizes but slightly reduced power when compared with responder rate. This reduced power is due to the appropriate reflection of adverse events by Epilepsy‐DOOR. Epilepsy‐DOOR provides a more holistic measure of anti‐seizure medication treatment effects, balancing relative benefits and harms, and has potential as a future endpoint in clinical trials in epilepsy.

## Graphical abstract

The Epilepsy‐DOOR is a new consumer‐codesigned endpoint for clinical trials of new pharmacotherapies for drug resistant focal epilepsy. Using data from previously completed phase 3 randomised controlled trials, this study demonstrates the Epilepsy‐DOOR detected similar effect sizes to responder rate, providing a more holistic measure of treatment effects which balances benefits and harms in the use of pharmacotherapies for the treatment of focal epilepsy.

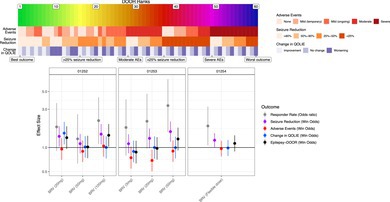

## 1 Introduction

Despite epilepsy being a multi‐faceted disease associated with a range of neurological, psychiatric and cognitive symptoms, clinical trials of anti‐seizure medications for drug resistant epilepsy are typically designed and powered to detect only reductions in seizure frequency—either as absolute reductions or responder rates (> 50% seizure reduction) without consideration for other measures. People with epilepsy and their treating neurologists regularly cite additional factors, including adverse events, cognitive deficits, psychiatric symptoms, cost and quality of life when considering the use of anti‐seizure medications in the management of their epilepsy [1, 2]. Increasingly, patients, doctors and pharmaceutical companies are becoming interested in more holistic, multidimensional outcomes for clinical trials. The development of new and different clinical trial endpoints that capture the multifaceted nature of epilepsy and concerns of patients, rather than just seizure response, is a major unmet need for the evaluation of new therapies. Recent efforts have focused on the development of patient reported outcome measures and other outcome measures to better capture the broad spectrum concerns of people with epilepsy and the potential benefits and harms of new epilepsy treatments [1, 2, 3]. However, these efforts have tended to be focused at demonstrating benefit of gene therapies in rare paediatric encephalopathies rather than more common conditions such as focal and generalised epilepsies. To provide a more multifaceted end point for epilepsy treatment trials, particularly those for disease modifying treatments where the goal is to improve epilepsy comorbidities as well as reduce seizure frequency, we recently adapted the desirability of outcome ranking (DOOR) for use in epilepsy [4]. This Epilepsy‐DOOR was codesigned with consumers, and included measures of seizures, adverse events and quality of life into a single measure that assesses the relative benefit versus harm of the treatment. The aim of this current study was to conduct a post hoc investigation of the Epilepsy‐DOOR as an outcome measure in clinical trials in epilepsy using previously undertaken Phase 3 trials that had collected the relevant data.

## 2 Methods

### 2.1 Data Selection

Data from three Phase 3 studies of double‐blind, randomised controlled trials of brivaracetam (BRV) treatment versus placebo in patients with drug resistant focal epilepsy (N01252, NCT00490035; N01253, NCT00464269; N01254, NCT00504881 [5, 6, 7]) were used in this analysis. All patients gave written informed consent prior to study entry. Full details on trial inclusion/exclusion criteria, IRB approvals and treatment periods can be found in the respective study reports [5, 6, 7]. Data were included for all participants for whom data on seizure frequency, adverse events and quality of life in epilepsy‐31 (QOLIE‐31) scores were available at baseline and end of treatment. In studies N01252 and N01253, participants were randomised to placebo or one of three doses of BRV: 20 mg, 50 mg, or 100 mg per day in study N01252 [6] and 5, 20 or 50 mg in study N01253 [5]. Study N01254 was a flexible‐dose study, and thus variable doses were reported in the participant population; as such, analysis in this present study compared BRV versus placebo [7]. Table 1 includes the numbers of participants in the trials and the final numbers included in the analyses. These studies were selected due to them being late phase placebo‐controlled, randomised controlled trials of an anti‐seizure medication in which the studies included measures of all three components of the Epilepsy‐DOOR—seizure frequency, adverse events and QOLIE‐31.

**TABLE 1:** Participant numbers in each study and group, including randomisation, inclusion in intention to treat (ITT) and per protocol (PP) analyses of original trials, and inclusion in the current analysis.

	N01252 (20 mg, 50 mg, 100 mg BRV, placebo)	N01253 (5 mg, 20 mg, 50 mg BRV, placebo)	N01254 (BRV, placebo)
Randomised	100, 100, 100, 99	99, 100, 102, 99	359, 121
Included in primary efficacy study (ITT)	99, 100, 100, 99	99, 100, 102, 99	359, 121
Completed study (PP)	93, 88, 94, 92	82, 93, 93, 93	323, 111
Included in current study	89, 90, 79, 86	85, 82, 82, 88	314, 106
Reason(s) for exclusion of subjects	Missing QOLIE‐31: 9, 8, 21, 13; and/or Missing AE data: 2, 2, 0, 0	Missing QOLIE‐31: 13, 18, 17, 11; and/or Missing AE data: 1, 0, 3, 0	Missing QOLIE‐31: 45, 15; and/or Missing AE data: 1,1

### 2.2 Epilepsy‐DOOR

**FIGURE 1:**
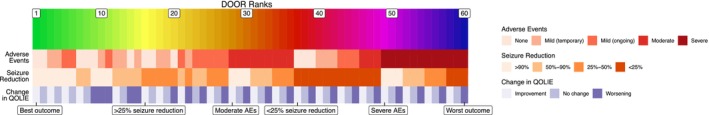
A schematic of the Epilepsy‐DOOR combinations.

The Epilepsy‐DOOR is a consumer codesigned clinical trial endpoint which combines changes in seizure frequency, adverse event severity and quality of life measures into a single outcome measure. The Epilepsy‐DOOR was co‐designed with, and applicable to adults with drug resistant focal epilepsy. Details on the development of the Epilepsy‐DOOR have been reported previously [4]. Each level of the Epilepsy‐DOOR consisted of multiple possible categorical outcomes: four for seizure frequency, five for adverse events and three for QOLIE‐31, resulting in 60 possible outcome ordinal ranks, with 1 being the best possible outcome (> 90% seizure reduction, no adverse events, improvement in quality of life) and 60 being the worst (< 25% seizure reduction, severe ongoing adverse events, worsening in quality of life). The Epilepsy‐DOOR can be broadly divided into 4 strata based on dominant outcome. The best outcomes are characterised by large reductions in seizure frequency, combined with minimal adverse events and no worsening of quality of life; the second strata dominated by moderate ongoing adverse events; the third strata by no (< 25%) reduction in seizure frequency; and the worst outcomes characterised by severe ongoing adverse events irrespective of benefit to seizures and quality of life measures. The full rank of combined outcomes can be found in our previous paper [4] and is summarised in Figure 1. Each participant was assigned an Epilepsy‐DOOR based on the combination of the measures across the duration of the respective studies. Change in seizure frequency was measured as the percentage change in weekly seizure frequency from baseline (averaged over 8 weeks) to end of treatment (averaged over 12 weeks); presence and severity of adverse events was defined as the most severe adverse event occurring during the treatment period; and change in QOLIE‐31 absolute change from baseline to end of treatment.

### 2.3 Statistical Analysis

Win odds were estimated for each treatment group compared to the control group in each study. The win odds estimate the likelihood that any randomly selected participant in each of the BRV groups will have a better outcome (i.e., “win”) over a randomly selected placebo‐treated participant [8]. Each participant in each of the BRV groups is compared to each participant in the placebo‐treated group, resulting in the overall win odds for that treatment group. A win odds of 1 indicates no benefit of treatment over placebo, with a win odds > 1 indicating the benefit of treatment over placebo.

Win odds of treatment benefit were estimated for % change in seizure frequency, adverse event severity, absolute change in QOLIE‐31 and Epilepsy‐DOOR to provide direct comparison between treatment groups for each group at each dose. Due to its binary nature, odds ratios were estimated for responder rate. 95% confidence intervals were reported for each measure and estimated *p* < 0.05 were considered significant.

For the purposes of descriptive comparisons, frequency of specific outcomes was compared using Chi squared tests. Further, due to this work being an illustrative re‐analysis of previously completed trials, despite overlapping doses between studies, comparison of doses across studies was not done due to the independence of the original trials.

## 3 Results

### 3.1 Epilepsy‐DOOR Shows Benefit of Brivaracetam Over Placebo

**FIGURE 2:**
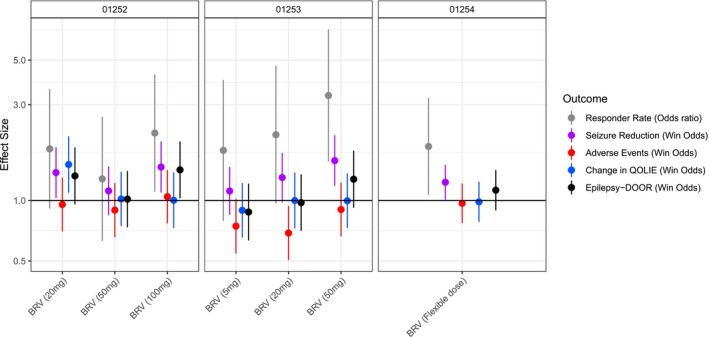
Effect sizes for benefit of brivaracetam treatment over placebo on outcome measures. Effect sizes are reported as win odds for all measures except the responder rate which is reported as odds ratios.

The Epilepsy‐DOOR demonstrated a numerical benefit of treatment with BRV over placebo in all the studies evaluated, albeit not statistically significant in a number of groups (Figure 2, Table 2). In the N01252 trial, the Epilepsy‐DOOR showed the benefit of 100 mg BRV over placebo (win odds 1.42, 95% CI 1.03, 1.97), which was also observed when measuring responder rate (odds ratio 2.17, 95% CI 1.11, 4.24). Lower doses did not show statistically significant benefit of BRV treatment when either Epilepsy‐DOOR or responder rate were measured. However, 20 mg BRV had a high odds ratio and win odds for responder rate (odds ratio 1.81, 95% CI 0.91, 3.59) and Epilepsy‐DOOR (win odds 1.33, 95% CI 0.96, 1.84), albeit not reaching statistical significance.

**TABLE 2:** Summary of effect sizes across each active treatment arm in N01252, N01253 and N01254.

	Responder rate	% change in seizure frequency	Adverse events	Change in QOLIE‐31	Epilepsy‐DOOR
*N01252*					
BRV 20 mg	1.81 (0.91, 3.59)***	1.38 (1.03, 1.84)*	0.96 (0.70, 1.31)	1.50 (1.09, 2.07)*	1.33 (0.96, 1.84)***
BRV 50 mg	1.28 (0.63, 2.61)	1.12 (0.85, 1.48)	0.90 (0.66, 1.22)	1.02 (0.75, 1.39)	1.02 (0.74, 1.4)
BRV 100 mg	2.17 (1.11, 4.25)*	1.47 (1.10, 1.97)*	1.05 (0.77, 1.42)	1.00 (0.73, 1.38)	1.42 (1.03, 1.97)*
*N01253*					
BRV 5 mg	1.78 (0.79, 3.99)	1.12 (0.85, 1.47)	0.75 (0.54, 1.02)***	0.89 (0.65, 1.22)	0.88 (0.63, 1.21)
BRV 20 mg	2.13 (0.96, 4.69)***	1.30 (0.98, 1.73)***	0.69 (0.51, 0.94)*	1.00 (0.73, 1.38)	0.98 (0.71, 1.35)
BRV 50 mg	3.33 (1.56, 7.11)**	1.58 (1.18, 2.11)**	0.90 (0.66, 1.23)	1.00 (0.73, 1.36)	1.28 (0.92, 1.77)
*N01254*					
BRV	1.86 (1.07, 3.24)*	1.23 (1.01, 1.51)*	0.97 (0.77, 1.22)	0.98 (0.78, 1.24)	1.13 (0.89, 1.42)

*Note:* Effect sizes are reported as win odds for all measures except responder rate, which is reported as odds ratios.

* *p* < 0.05.

** *p* < 0.01.

*** *p* < 0.1 compared to placebo.

In the N01253 trial, 50 mg BRV (but not the other doses) demonstrated greater responder rates than placebo (odds ratio 3.33, 95% CI 1.56, 7.11); however, the Epilepsy‐DOOR did not demonstrate statistically significant benefit of 50 mg BRV over placebo (win odds 1.28, 95% CI 0.92, 1.77). The 20 mg group had a higher responder rate (odds ratio 2.13, 95% CI 0.96, 4.69) and % change in seizure frequency over placebo (win odds 1.30, 95% CI 0.98, 1.73) which did not reach statistical significance; however, adverse events were worse than placebo in the 20 mg group (win odds 0.69, 95% CI 0.51, 0.94) and thus the Epilepsy‐DOOR showed no benefit over placebo (win odds 0.98, 95% CI 0.71, 1.35). The 5 mg group also demonstrated non‐significantly worse adverse events (win odds 0.75, 95% CI 0.54, 1.02), despite no benefit on any seizure outcome measures.

The Epilepsy‐DOOR did not demonstrate positive outcomes in the flexible dosing study (N01254, win odds 1.13, 95% CI 0.89, 1.42), unlike responder rate and % change in seizure frequency which both showed benefit of BRV over placebo (odds ratio 1.85, 95% CI 1.07, 3.24, win odds 1.23, 95% CI 1.01, 1.51).

### 3.2 Epilepsy‐DOOR Outcomes Were Highly Variable Across Studies and Groups

**FIGURE 3:**
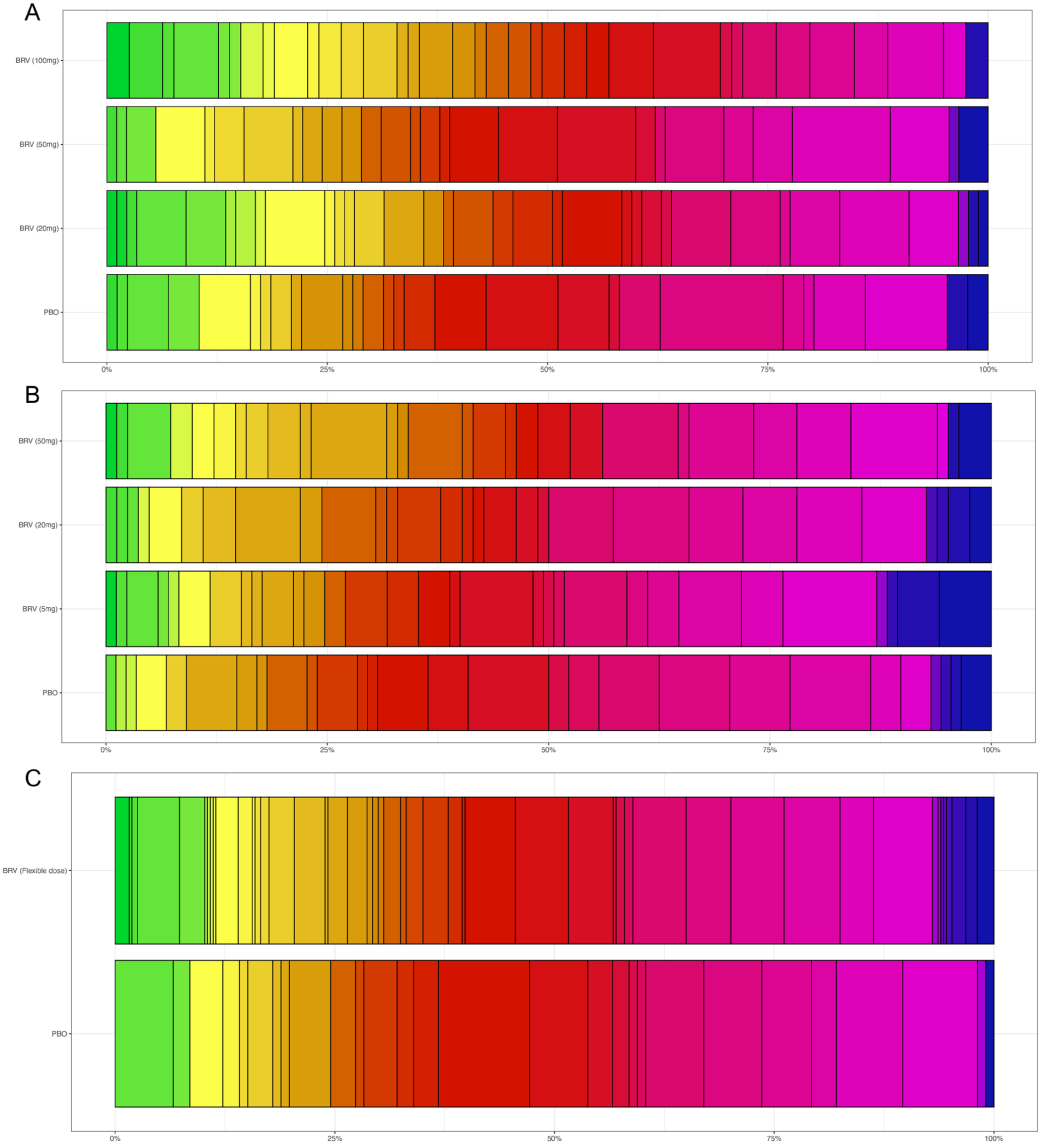
Frequency distribution of individual DOORs in each group/study. Top N01252, Middle N01253, Bottom N01254, with individual rows representing doses of brivaracetam (high to low) followed by placebo. Each colour represents an individual rank, with green being the best possible outcomes, through to blue being the worst outcomes. The width of each bar represents the proportion of participants who were given that specific rank. See Figure 1 for details of how variables are combined to give Epilepsy‐DOOR.

The Epilepsy‐DOOR showed variable outcomes across the groups and participants. Figure 3 shows the distribution of DOORs across the different groups the studies, demonstrating a wide range of outcomes across the whole study group. Table 3 summarises the proportions of subjects in each stratum of the Epilepsy‐DOOR. In N01253 this shows a clear dose response effect with increasing proportions in the active treatment groups in strata 1 (18.2% in placebo group, increasing to 32.9% in the 50 mg BRV group). Conversely, in N01252 proportions in the first strata were more varied, being 26.7% and 28.9% in the placebo and 50 mg BRV group, but 38.2% and 41.8% in the 20 mg and 100 mg BRV groups. Proportion of subjects in strata 1 in N01254 were similar in placebo and active groups, 24.5% and 29.3% respectively. At the opposite end of the scale, proportions in strata 4 also varied considerably between studies and groups. In N01252, proportions were low and similar across all groups (2.5%–4.7%), whereas in N01253, the proportion of subjects in strata 4 greater overall ranging from 6.1%–12.9% across the groups. The proportion in strata 4 was also larger in the BRV group (7.0%) than placebo (1.9%) in N01254.

**TABLE 3:** Breakdown of proportion of participants in each broad Epilepsy‐DOOR stratum per treatment group.

	Strata 1 (> 25% seizure reduction, minimal AEs)	Strata 2 (> 25% seizure reduction, moderate AEs)	Strata 3 (< 25% seizure reduction)	Strata 4 (severe AEs)
*N01252*				
Placebo	23/86 (26.7%)	9/86 (10.5%)	50/86 (58.1%)	4/86 (4.7%)
BRV 20 mg	34/89 (38.2%)**	12/89 (13.5%)	40/89 (44.9%)	3/89 (3.4%)
BRV 50 mg	26/90 (28.9%)	9/90 (10%)	51/90 (56.7%)	4/90 (4.4%)
BRV 100 mg	33/79 (41.8%)*	10/79 (12.7%)	34/79 (43.0%)	2/79 (2.5%)
*N01253*				
Placebo	16/88 (18.2%)	11/88 (12.5%)	55/88 (62.5%)	6/88 (6.8%)
BRV 5 mg	21/85 (24.7%)	9/85 (10.6%)	44/85 (51.8%)	11/85 (12.9%)
BRV 20 mg	20/82 (24.4%)	14/82 (17.1%)	42/82 (51.2%)	6/82 (7.3%)
BRV 50 mg	27/82 (32.9%)*	11/82 (13.4%)	39/82 (47.6%)**	5/82 (6.1%)
*N01254*				
Placebo	26/106 (24.5%)	13/106 (12.3%)	65/106 (61.3%)	2/106 (1.9%)
BRV	92/314 (29.3%)	33/314 (10.5%)	167/314 (53.2%)	22/314 (7.0%)*

*Note:* Chi‐squared tests measured proportion in each stratum.

* *p* < 0.05.

** *p* < 0.1 compared to placebo.

**FIGURE 4:**
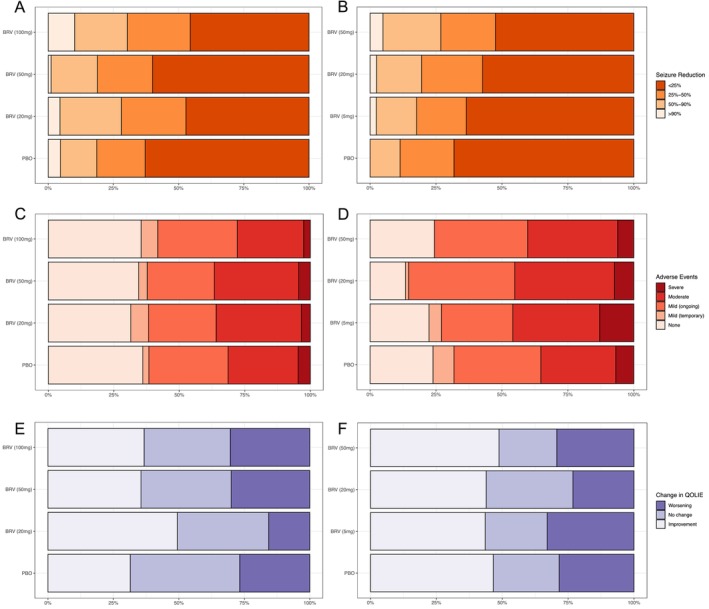
Breakdown of individual components of Epilepsy‐DOOR for all participants in the N01252 (left) and N01253 (right). Top row: reduction in seizures, middle row: presence and severity of adverse events, bottom row: change in QOLIE.

When examining the distributions of outcomes for the different variables more closely, similar distributions of outcomes can be seen for QOLIE and AE measures (Figure 4), with differences in seizure frequency change categories between groups. For example, in N01252, 64.5% of participants in the placebo group had minimal (< 25% reduction) in seizure frequency, compared to 49.5%, 59% and 48% in the 20, 50 and 100 mg BRV groups. This was also observed in N01253, with 65.7% of participants in the placebo group showing minimal seizure reduction, compared to 62.6%, 55% and 48% in the 5, 20 and 50 mg groups, and in N01254 was 63.6% in the placebo group and 56.3% in the BRV group. Differences in greater seizure reductions (combined > 50% and > 90% reduction) were also observed across doses, including 17.2% of placebo participants, and 27.3%, 21% and 31% of participants in the 20, 50 and 100 mg BRV groups in N01252; 11.1% of placebo participants and 18.2%, 21% and 29.4% of participants in the 5, 20, and 50 mg BRV groups in N01253, and 14.9% and 24.4% in the placebo and BRV groups in N01254.

Despite these demonstrations of greater impact on seizure frequency with higher doses of BRV, differences in adverse events and quality of life measures did impact overall Epilepsy‐DOOR outcomes, as seen in the 20 mg group in N01252, where improved QOLIE‐31 scores contributed to overall Epilepsy‐DOOR score, and in N01253, where worse adverse events in the 20 mg group mediated the effects of seizure control on the overall Epilepsy‐DOOR score (Table 2, Figures 3 and 4).

## 4 Discussion

This study demonstrates the use of a new outcome measure of clinical treatment trials in epilepsy, the Epilepsy‐DOOR. The main findings were: (1) Using data from the pivotal phase 3 clinical trials of BRV as an adjunctive therapy in drug resistant epilepsy, the Epilepsy‐DOOR was able to demonstrate the benefit of BRV treatment over placebo in some but not all treatment groups, (2) Epilepsy‐DOOR has similar effect sizes, but diminished power when compared with responder rate, (3) the diminished power was due to the presence and severity of adverse events balanced against the reduction in seizure frequency, (4) the Epilepsy‐DOOR provides a more holistic measure of benefit and harms associated with trials of epilepsy treatments.

In the N01252 trial the Epilepsy‐DOOR showed similar patterns of significant difference between the BRV and placebo treatments to that seen with responder rate, with only the highest dose (100 mg) demonstrating benefit over placebo. This is in agreement with the findings of the original trial [6], wherein 100 mg, but not 50 or 20 mg, showed greater percentage reduction in seizures and responder rate (> 50% reduction in seizure frequency) than placebo treatment [6]. Conversely, in the N01253 trial, the Epilepsy‐DOOR did not show significant benefit of BRV treatment at any dose, despite responder rate showing a significant effect at 50 mg (but not at other doses). The original trial also only found benefit in percentage change and responder rate highest dose group [5]. The Epilepsy‐DOOR did not show benefit in the N01254 flexible dose study, similar to the original study results, in which percentage change in seizure frequency was not different between BRV and placebo treated participants, but responder rate did show benefit over placebo [7]. We have demonstrated that it is the interplay between seizure reduction and adverse events that drives the differences in the Epilepsy‐DOOR outcome between doses and studies. With the N01252 trial, the frequency and severity of adverse events was similar across groups; therefore, the benefit measured by the Epilepsy‐DOOR was driven by a reduction in seizures. However, with the N01253, the adverse events had a significant negative effect, particularly at the lower doses, mitigating the effect of a reduction in seizures; thus, no overall benefit over placebo was measured at any dose on the Epilepsy‐DOOR. It is important to note that while at the highest BRV dose (50 mg) in the N01253 and N01254 trials the effect size for both seizure responder rate and the Epilepsy‐DOOR were of a similar magnitude, the lack of statistical significance for the Epilepsy‐DOOR was due to the large confidence intervals and the slightly diminished power when using the Epilepsy‐DOOR over seizure responder rate alone.

Whilst the Epilepsy‐DOOR was not able to demonstrate benefit of BRV treatment in all treatment groups where benefit was shown when measuring seizure responder rate alone, the multiple aspects of the outcome measure may better reflect the clinical picture when a patient and their doctor are considering the benefits versus adverse effects of an anti‐seizure medication. As a holistic outcome measure, the Epilepsy‐DOOR better captures the multifaceted nature of epilepsy and the effects of epilepsy treatments, providing patients and doctors with a measure of real‐world relevance that more closely reflects the clinical setting, where patients consider seizure burden alongside other concerns when discussing treatment options with their epileptologist.

The DOOR was initially developed for the assessment of benefits versus harms of antibiotic treatments in an inpatient setting with 8 possible ranks [9]. Similarly, the win ratio, a relatively new approach, has primarily been used in studies using hierarchical composites, many of which consist of multiple time to event measures [10], given these considerations, ties are very common when applying the win ratio. To overcome interpretation problems in the presence of a high frequency of ties the win ratio has been adapted to win odds which accounts for, rather than excludes ties, when measuring study outcomes [8]. The Epilepsy‐DOOR, which was co‐designed with people with epilepsy and other consumers, combines three multi‐level measures producing 60 individual outcome ranks. This highly granular measure minimises the likelihood of ties, which can occur commonly in “time to event” trials, particularly when events occur at low frequency overall [8, 10]. The Epilepsy‐DOOR was developed for evaluation of new (primarily adjunctive) treatments for adults with drug resistant focal epilepsy; however, it could be adapted for studies of first line therapies or other epilepsy populations (paediatric epilepsies, new‐onset epilepsy) using the same consumer co‐design approach described in our development paper [4]. Such adaptations could include time to event rather than change in seizure frequency and other measures (mood, cognitive and behavioural symptoms) with greater relevance to different populations. Other elements, such as medication burden, treatment satisfaction, and safety outcomes could also be considered when adapting the Epilepsy‐DOOR to other patient groups.

The Epilepsy‐DOOR was developed for use as the primary outcome measure in our current clinical trial of sodium selenate as a potential disease‐modifying treatment for patients with chronic drug resistant mesial temporal lobe epilepsy (SeLECT [11], ACTRN12623000446662). This trial also includes ‘standard’ outcome measures (seizure frequency, interictal epileptiform discharge frequency, quality of life, cognition, neuropsychiatric symptoms) and statistical analysis techniques (linear mixed effects models) as secondary/exploratory outcomes. As part of this trial, and another interventional study being run at our centre (KetoCoach, ACTRN12624000486527), we will prospectively evaluate the Epilepsy‐DOOR as an outcome measure against these established secondary endpoints.

Evaluation in clinical settings could also support use of the Epilepsy‐DOOR. In centres with registries where seizure and other epilepsy related measures (such as the QOLIE) are recorded in a standardised manner, Epilepsy‐DOOR outcomes could be measured when changing treatments (including neuromodulation and surgery), enabling benefits and harms to be assessed in real world clinical situations and providing real world usage of this outcome measure.

It remains to be seen how the Epilepsy‐DOOR will be received by the regulatory authorities, such as the US Food and Drug Administration, The European Medicine Agency or the Australian Therapeutic Goods Administration, for licencing of new epilepsy therapies. The FDA's patient reported outcome measures (PROMs) guidance document [12] provides a framework for development, validation and use of PROMs as trial endpoints, the Epilepsy‐DOOR could be validated using a similar process.

### 4.1 Limitations

Whilst this work has demonstrated the use of the Epilepsy‐DOOR as an outcome measure for randomised controlled trials, there are several limitations. Firstly, we have only investigated a single anti‐seizure medication, BRV. This was due to the availability of trial data in drug resistant focal epilepsy which included the QOLIE in its outcome measures; other recent randomised controlled trials have not included the QOLIE as an outcome measure, with data from older trials which do include the QOLIE not accessible. Secondly, this is a retrospective analysis of previously collected data; however, the Epilepsy‐DOOR will be used in our ongoing clinical trial providing prospective investigation of the Epilepsy‐DOOR as a potential future outcome measure.

## 5 Conclusion

The Epilepsy‐DOOR is a promising multifaceted outcome measure for use as an endpoint in interventional clinical trials in drug resistant focal epilepsy in adults that assess both the benefits and harms of the treatment. Future studies will provide further validation for its use against established clinical trial endpoints.

## Author Contributions

L.V. and L.C. conceived of the study. All authors contributed to study design. L.V. and H.J. collated and analysed the data. L.V. wrote the manuscript. All authors reviewed and approved the final version.

## Funding

This research is supported by an Australian Government MRFF grant (GNT2023250) to the authors and an NHMRC Investigator Grant to TJO (APP1176426).

## Ethics Statement

Data used in this manuscript came from previously completed trials as detailed in the data availability statement below. All patients gave written informed consent prior to study entry. Full details on IRB approvals can be found in the respective study reports [5, 6, 7].

## Conflicts of Interest

The authors declare no conflicts of interest.

## Data Availability

The data that support the findings of this study are available from UCB, made available via Vivli Inc. Restrictions apply to the availability of these data, which were used under licence for this study. Data requests may be made to Vivli Inc. with the permission of UCB. R codes used for statistical analyses will be available to qualified investigators by contacting the corresponding author.
